# Subpopulations of Stressed Yersinia pseudotuberculosis Preferentially Survive Doxycycline Treatment within Host Tissues

**DOI:** 10.1128/mBio.00901-20

**Published:** 2020-08-04

**Authors:** Jasmine Ramirez Raneses, Alysha L. Ellison, Bessie Liu, Kimberly M. Davis

**Affiliations:** a W. Harry Feinstone Department of Molecular Microbiology and Immunology, Johns Hopkins Bloomberg School of Public Health, Baltimore, Maryland, USA; University of North Carolina at Chapel Hill; Washington University School of Medicine

**Keywords:** antibiotic diffusion, doxycycline, fluorescent reporter, systemic infection, tetracycline derivatives, *Yersinia*

## Abstract

Severe systemic bacterial infections result in colonization of deep tissues, which can be very difficult to eliminate with antibiotics. It remains unclear if this is because antibiotics are not reaching inhibitory concentrations within tissues, if subsets of bacteria are less susceptible to antibiotics, or if both contribute to limited treatment efficacy. To detect exposure to doxycycline (Dox) present in deep tissues following treatment, we generated a fluorescent transcriptional reporter derived from the *tet* operon to specifically detect intracellular tetracycline exposure at the single bacterial cell level. Dox exposure was detected in the spleen 2 h after intraperitoneal injection, and by 4 h postinjection, this treatment resulted in a significant decrease in viable Yersinia pseudotuberculosis bacteria in the spleen. Nitric oxide-stressed bacteria preferentially survived treatment, suggesting that stress was sufficient to alter Dox susceptibility. Many bacteria (∼10%) survived a single dose of Dox, and the antibiotic accumulated at the periphery of microcolonies to growth inhibitory concentrations until 48 h posttreatment. After this time point, antibiotic concentrations decreased and bacterial growth resumed. Dox-treated mice eventually succumbed to the infection, albeit with significantly prolonged survival relative to that of untreated mice. These results indicate that Dox delivery by intraperitoneal injection results in rapid diffusion of inhibitory concentrations of antibiotic into the spleen, but stressed cells preferentially survive drug treatment, and bacterial growth resumes once drug concentrations decrease. This fluorescent reporter strategy for antibiotic detection could easily be modified to detect the concentration of additional antimicrobial compounds within host tissues following drug administration.

## Abstract (precis)

Bacterial infections are very difficult to treat when bacteria spread into the bloodstream and begin to replicate within deep tissues, such as the spleen. Subsets of bacteria can survive antibiotic treatment, but it remains unclear if this survival is because of limited drug diffusion into tissues, or if there are changes within the bacteria, promoting survival of some bacterial cells. Here, we have developed a fluorescent reporter to detect doxycycline (Dox) diffusion into host tissues, and we show that Dox impacts the bacterial population within hours of administration and inhibits bacterial growth for 48 h. However, bacterial growth resumes when antibiotic concentrations decrease. Subsets of bacteria, stressed by the host response to infection, survive Dox treatment at a higher rate. These results provide critical information about the dynamics that occur within deep tissues following antibiotic administration and suggest that subsets of bacteria are predisposed to survive inhibitory concentrations of antibiotic before exposure.

## INTRODUCTION

Bacterial infections are exceedingly difficult to treat with antibiotics when bacteria spread systemically into deep tissues. It is thought that this difficulty arises because the antibiotic cannot accumulate to inhibitory concentrations within these tissue sites, so bacteria are exposed to subinhibitory concentrations of the drug. It has been difficult to determine the antibiotic concentration present within deep tissues, and many studies have relied on serum concentrations to determine whether tissue contain inhibitory levels (1, 2). Recently, mass spectrometry-based assays have shown differential diffusion of tuberculosis drugs across granulomas (3). Biofilm structures also limit antibiotic diffusion into the center of bacterial communities. Both granulomas and biofilms contain stressed, slow-growing bacterial subpopulations at the center of the bacterial community, and it is thought that inhibitory concentrations of antibiotic cannot reach these cells, so they remain following treatment (4–8). Mass spectrometry could be used to better understand antibiotic diffusion within tissues but requires specialized technology for tissue processing, data collection, and analysis. Bacterial fluorescent reporters offer a cost-effective alternative that can detect spatiotemporal changes in antibiotic concentration across individual bacterial cells following treatment.

Slow-growing bacterial cells are known to be less susceptible to antibiotics, but it remains unclear which pathways promote slowed bacterial growth within host tissues (9, 10). Nutrient limitation may reduce metabolic activity and subsequently reduce antibiotic susceptibility (4, 11, 12). Host immune cell subsets can promote the formation of slow-growing bacteria within host tissues (13, 14), but it remains unclear if antimicrobial compounds such as reactive oxygen species (ROS) or reactive nitrogen species (RNS) contribute to slowed growth (15, 16). Recently, it was shown that ROS exposure was sufficient to reduce the rifampin susceptibility of Staphylococcus aureus during spleen infection (17). However, it remains unclear if bacterial cells responding to ROS preferentially survived drug treatment. Expression of virulence factors, and specifically type III secretion system (T3SS) expression, has been linked to slowed growth and decreased antibiotic susceptibility in Salmonella species, but this link has not been made within host tissues (18, 19).

Yersinia pseudotuberculosis replicates within deep tissue sites to form clonal extracellular clusters of bacteria called microcolonies (or pyogranulomas) (20–22). Within the spleen, neutrophils are recruited to sites of bacterial replication and directly contact bacteria at the periphery of microcolonies. Peripheral bacteria respond to neutrophil contact by expressing high levels of the type III secretion system (T3SS), which promotes translocation of T3SS effector proteins into neutrophils and inhibits phagocytosis. A layer of monocytes is recruited immediately around the layer of neutrophils. Recruited monocytes develop characteristics of dendritic cells and macrophages (20–22) and express inducible nitric oxide synthase (iNOS), which produces antimicrobial nitric oxide (NO) that diffuses toward the microcolony. Bacteria at the periphery of the microcolony respond to NO by expressing the NO-detoxifying gene, *hmp* (22). *hmp* expression at the periphery prevents NO diffusion into the center of microcolonies, establishing an example of cooperative behavior in which the peripheral bacteria protect the interior bacteria from the antimicrobial action of NO (22, 23). It remains unclear if the protective expression of *hmp* by peripheral cells comes at a fitness cost, and if this stress response is sufficient to alter the antibiotic susceptibility of this subpopulation.

The genetic tools available for Y. pseudotuberculosis allow us to easily construct fluorescent reporters to detect changes in the host environment at the single-bacterium level (22–24). Here, we introduce a tetracycline-responsive fluorescent reporter based on the *tet* operon that enables detection of the diffusion of tetracycline derivatives, including doxycycline (Dox), within host tissues. Dox was chosen for these studies because it is a relevant bacteriostatic antibiotic for treatment of Yersinia infection (25–27), but similar reporters could also be generated for other classes of antibiotics. This tool allows us to determine if individual bacteria are differentially exposed to antibiotics during drug treatment within mouse tissues and to more closely approximate antibiotic concentrations within host tissues.

## RESULTS

### Characterizing the doxycycline-responsive reporter.

Bacterial infections of deep tissues are exceedingly difficult to treat, and it is thought that this is because the concentration of antibiotic does not reach inhibitory levels within deep tissue sites. Here, we establish a reporter system to detect Y. pseudotuberculosis antibiotic exposure within deep tissues, using an antibiotic prescribed to treat infection, doxycycline (Dox), to better understand what occurs within host tissues after antibiotic administration. To detect antibiotic diffusion and exposure, we constructed a tetracycline-responsive transcriptional reporter within a low-copy plasmid (pMMB67EH), and transformed the reporter into wild-type (WT) Y. pseudotuberculosis. The reporter contains a constitutively expressed *tetR* gene whose gene product, TetR, binds to DNA and represses expression of P*_tetA_*::*mCherry* in the absence of tetracycline derivatives. In the presence of tetracyclines, drug binding to TetR relieves repression, and transcription of *mCherry* occurs (see Fig. S1A in the supplemental material).

10.1128/mBio.00901-20.1 FIG S1 Characterizing the doxycycline (Dox)-responsive reporter. (A) Tetracycline responsive reporter schematic. In the absence of tetracyclines (−Tet), the repressor (TetR) binds at the *tetO* sequence to inhibit expression of *mCherry*, inserted downstream of the P*_tetA_* promoter. In the presence of tetracyclines (+Tet), TetR repression is relieved and *mCherry* is expressed. (B) Growth curve of the P*_tetA_*::*mCherry* strain with the indicated doses of Dox. Optical density (*A*_600_) is measured over time (h). Mean and error of three biological replicates are shown. (C) Reporter expression during growth curve, detected with 560-nm excitation/610-nm emission, expressed as fold increase of mCherry signal relative to that in untreated cells. Median and range depicted; four biological replicates. (D) Reporter expression within individual bacterial cells detected by fluorescence microscopy after 4 h of treatment, expressed as fold increase in mean mCherry signal/cell relative to the average signal of untreated cells. Experiment performed in triplicate; one representative is shown with medians. Statistics: two-way ANOVA, Tukey’s multiple-comparison test in panels B and C and Kruskal-Wallis with Dunn’s multiple-comparison test in panel D. ****, *P* < 0.0001. Download FIG S1, TIF file, 1.0 MB. Copyright © 2020 Ramirez Raneses et al. 2020 Ramirez Raneses et al. This content is distributed under the terms of the Creative Commons Attribution 4.0 International license.

To determine if the P*_tetA_* reporter can be used to quantify the amount of Dox exposure within individual bacteria, we treated liquid cultures of the reporter strain with increasing doses of Dox. Bacterial growth (*A*_600_) and mCherry fluorescence were quantified in a microplate reader. Treatment with 1 μg/ml Dox resulted in growth inhibition consistent with published MIC levels for *Yersinia* and close to the maximum Dox concentration range that can be obtained within mouse and human serum (Fig. S1B) (2, 28–30). Reporter signal was detected with 0.01 μg/ml Dox and significantly increased with 0.1 μg/ml Dox (Fig. S1B and C). Exposure to 1 μg/ml resulted in decreased reporter expression, likely due to ribosomal inhibition by Dox. Reporter signal was first detected after 2 h of antibiotic exposure and significantly increased after 4 h of exposure (Fig. S1C).

Plate reader measurements give averaged, population-level readings; to detect heterogeneity across individual bacterial cells, reporter signal quantified at the single-cell level by fluorescence microscopy. Single-cell quantification also showed that 0.01 μg/ml Dox increased reporter signal, 0.1 μg/ml Dox significantly increased signal, and 1 μg/ml treatment decreased reporter signal within individual cells (Fig. S1D). Cells within each treatment group had distinct levels of fluorescent signal in response to Dox, suggesting that this reporter will be a useful tool for determining antibiotic exposure levels. Collectively, these results confirm the reporter responds to Dox and can be used to detect antibiotic exposure within individual bacterial cells.

### Doxycycline differentially diffuses across microcolonies.

**FIG 1:**
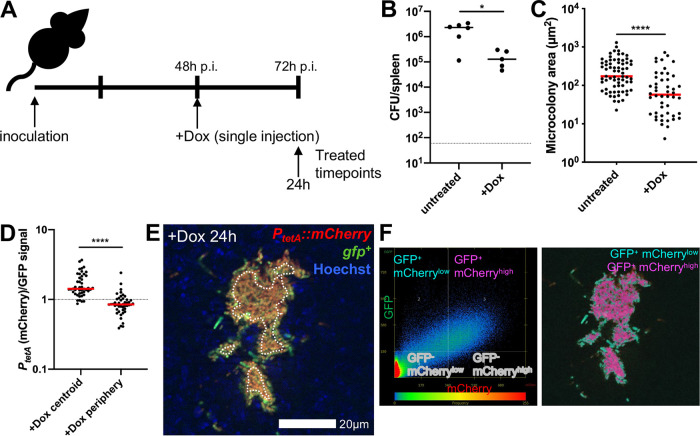
Doxycycline differentially diffuses across microcolonies. (A) Timeline for mouse infections. C57BL/6 mice were inoculated intravenously (i.v.) with 10^3^ green fluorescent protein-positive (GFP^+^) P*_tetA_*::*mCherry* Y. pseudotuberculosis. Mice were treated at 48 h postinoculation (h p.i.) with 40 mg/kg Dox (Dox) in 100 μl PBS, administered intraperitoneally (i.p.), and spleens were harvested at the indicated time points to quantify CFU or process tissue for fluorescence microscopy. (B) CFU/spleen quantification from treated (+Dox, 24 h) or untreated mice (72 h). Dots indicate individual mice. (C) Fluorescence microscopy of spleens to quantify microcolony area (μm^2^) from treated (+Dox) or untreated mice, based on GFP signal. Dots indicate individual microcolonies. (D) Reporter signals were quantified from peripheral and centroid cells within treated microcolonies in the spleen, and reporter signal was divided by GFP. (E) Representative image; cells outside dotted white line are defined as peripheral. (F) Colocalization analysis of image shown in panel E, to show differential reporter signal by pseudocolor. Dot plot depicts signal intensity for each pixel within the image. Quadrants were set using GFP^+^ signal to distinguish bacteria from surrounding tissue; within this gate, the 66% highest mCherry pixels are shown as mCherry^high^ (fuchsia), and the lowest 34% mCherry pixels are shown as mCherry^low^ (teal). Mouse infections were completed in three distinct experiments, and animal numbers depicted in Fig. 2B were processed for fluorescence microscopy (shown in Fig. 2C to F); medians are shown. Statistics: Mann-Whitney in panels B and C and Wilcoxon matched pairs in panel D. *, *P* < 0.05; ****, *P* < 0.0001.

To determine if Dox can diffuse into microcolonies and impact bacterial growth, we infected C57BL/6 mice intravenously with green fluorescent-protein-positive (GFP^+^) P*_tetA_*::*mCherry* Y. pseudotuberculosis and injected mice intraperitoneally with 40 mg/kg Dox at 48 h postinoculation (h p.i.). The 48-h time point p.i. was chosen to allow microcolonies to initially replicate and form distinct bacterial subpopulations prior to antibiotic treatment (22); intraperitoneal administration of 40 mg/kg Dox should result in inhibitory levels of Dox in the serum (1 to 4 μg/ml) within hours of injection (31, 32). Spleens were harvested at 24 h posttreatment to quantify CFU, microcolony areas, and reporter expression (Fig. 1A). Dox treatment resulted in significantly fewer CFU relative to those in untreated mice; however, ∼10^5^ CFU remained in the spleen 24 h after treatment (Fig. 1B). The area of microcolonies was also significantly lower following 24 h of Dox treatment compared to that in untreated mice, suggesting that antibiotic treatment either inhibited growth of microcolonies or promoted elimination of a subset of bacteria (Fig. 1C).

To determine if Dox diffused into microcolonies, the Dox-responsive reporter signal was compared to that of a constitutively expressed GFP. Reporter signal was significantly higher at the centroid of microcolonies compared to that at the periphery (outside dotted white line), suggesting that Dox diffused into the microcolony and that there was differential diffusion of Dox across microcolonies at 24 h posttreatment (Fig. 1D and E). Colocalization analysis was used to highlight differences in mCherry signal intensity across microcolonies and shows high mCherry reporter expression in the middle of microcolonies (fuchsia) and low mCherry signal within peripheral cells (teal) (Fig. 1F). Decreased reporter signal at the periphery may indicate that heightened levels of antibiotic accumulate at this location, limiting the translational activity of peripheral cells. However, it could also suggest antibiotic accumulation at the centroid of microcolonies and lower concentrations at the periphery.

### Tetracycline derivatives accumulate at the periphery of microcolonies.

**FIG 2:**
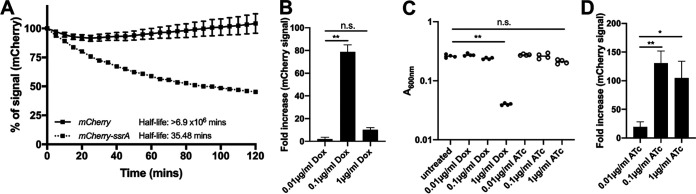
Anhydrotetracycline can be used to approximate antibiotic exposure without significant growth inhibition. (A) One-phase exponential decay equations calculate the half-life of mCherry and destabilized *mCherry-ssrA*. Fluorescence expression was induced with isopropyl-β-d-thiogalactopyranoside (IPTG), and kanamycin was then added to inhibit further translation. Fluorescence was detected over time (minutes). Data represent percentage of fluorescent signal intensity relative to time 0 (kanamycin addition). Mean and standard deviation of 6 biological replicates shown. (B) Unstable fluorescent reporter (P*_tetA_*::*mCherry-ssrA*) expression with the indicated doses of Dox, treated for 4 h at 37°C; fluorescence detected by plate reader. Fold increase relative to untreated cells; 4 biological replicates shown with median and range. (C) Optical density (*A*_600_) at 4 h posttreatment with the indicated doses of Dox and ATc. Dots represent individual cultures. (D) Unstable fluorescent reporter expression with the indicated doses of ATc, treated for 4 h at 37°C; fluorescence detected by plate reader. Fold increase relative to untreated cells; 5 biological replicates shown with median and range. Statistics: Kruskal-Wallis with uncorrected Dunn’s. *, *P* < 0.05; **, *P* < 0.01; n.s., not significant.

Our initial experiments in Fig. 1 utilized a reporter constructed with a stable mCherry fluorescent protein, which means reporter signal could accumulate over the course of the experiment. To confirm that reporter signal represents current responses to Dox, we generated a fluorescent reporter construct with a destabilized mCherry fluorescent protein. The destabilized mCherry within this construct has a *ssrA* tag (*mCherry-ssrA*), which targets mCherry for proteasomal degradation and quickens protein turnover (33, 34). *mCherry-ssrA* has a half-life of 35.5 min (Fig. 2A), which allows detection of recent responses to the antibiotic. Liquid cultures of the destabilized reporter strain were treated with increasing doses of Dox, and fluorescent reporter expression was quantified at 4 h posttreatment in a plate reader. Reporter expression patterns for the destabilized reporter were similar to those of the stable reporter at 4 h; destabilized fluorescent signal was maximal with 0.1 μg/ml Dox (Fig. 2B).

To determine if the Dox concentrations at the periphery of microcolonies are reaching levels that inhibit translation, we performed experiments with the inactive tetracycline derivative anhydrotetracycline (ATc). ATc can bind TetR and derepress the *tet* operon; however, ATc has an ∼35× lower binding affinity for E. coli ribosomes than that of Dox (35, 36). As it remained unclear if high concentrations of ATc impact Y. pseudotuberculosis growth, we treated liquid cultures of the reporter strain with increasing doses of ATc and detected growth and fluorescence with a microplate reader. Growth was significantly reduced with 1 μg/ml Dox but was not significantly impacted by 1 μg/ml ATc (Fig. 2C). Reporter signal was detected with 0.01 μg/ml ATc and was significantly increased with 0.1 μg/ml ATc. ATc at 1 μg/ml also resulted in high levels of reporter expression, in contrast to results with Dox, suggesting that ATc does not significantly inhibit ribosomal activity at this dose (Fig. 2D). Similar patterns were seen using flow cytometry to detect fluorescent reporter signals (see Fig. S2 in the supplemental material). The high level of reporter expression with 1 μg/ml ATc suggested that we could use ATc treatment to determine if peripheral bacteria are exposed to higher levels of antibiotic than the centroid, as increasing doses of ATc result in a heightened reporter signal.

10.1128/mBio.00901-20.2 FIG S2 Doxycycline-inhibited cells have low levels of mCherry signal. The P*_tetA_*::*mCherry* strain was exposed to the indicated doses of doxycycline (Dox) or anhydrotetracycline (ATc) during growth in LB at 37°C. mCherry reporter expression within individual bacterial cells was detected by flow cytometry after 4 h of treatment, and is expressed as the fold increase in mCherry mean fluorescent intensity (MFI) relative to the signal from untreated cells. Experiment performed in duplicate; biological replicates shown with medians. Download FIG S2, TIF file, 0.7 MB. Copyright © 2020 Ramirez Raneses et al. 2020 Ramirez Raneses et al. This content is distributed under the terms of the Creative Commons Attribution 4.0 International license.

**FIG 3:**
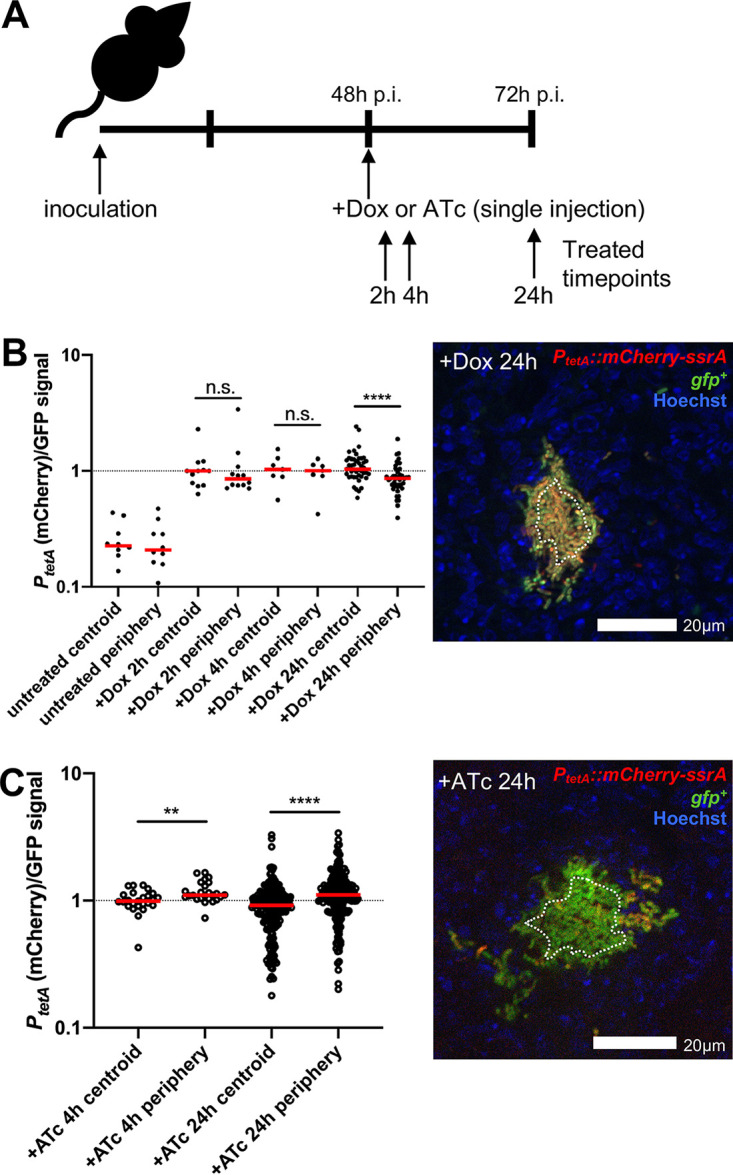
Tetracycline derivatives accumulate at the periphery of microcolonies. (A) Timeline for mouse infections. C57BL/6 mice were inoculated i.v. with 10^3^ GFP^+^ P*_tetA_*::*mCherry-ssrA* Y. pseudotuberculosis. Mice were treated at 48 h p.i. with 40 mg/kg Dox or 40 mg/kg ATc or left untreated (48 h), and spleens were harvested at the indicated time points posttreatment to process for fluorescence microscopy. (B) Reporter signal was quantified relative to GFP within peripheral and centroid cells, within microcolonies following Dox treatment. Dotted line indicates value of 1 (equivalent fluorescent intensity). Dots indicate individual microcolonies. Experiments performed on 2 days (4 to 5 mice/group); a representative image is shown (cells outside dotted white line are considered periphery). (C) Reporter signal was quantified within peripheral and centroid cells within microcolonies following ATc treatment. Experiments performed on two distinct days (5 to 6 mice/group); a representative image is shown (cells outside dotted white line are considered periphery). Medians are shown. Statistics in panels B and C: Wilcoxon matched pairs. **, *P* < 0.01; ****, *P* < 0.0001; n.s., not significant.

We then used the destabilized reporter strain in combination with Dox or ATc treatment to determine the concentration of antibiotics within microcolonies, to determine when antibiotic exposure occurs after treatment, and to confirm that spatial differences in reporter signal are not due to accumulation of stable fluorescent protein. Mice were infected intravenously with the GFP^+^ P*_tetA_*::*mCherry-ssrA* strain and treated at 48 h p.i., and splenic tissue was harvested at 2, 4, and 24 h posttreatment (Fig. 3A). Reporter expression was significantly higher at the centroid of microcolonies after 24 h of Dox treatment, suggesting that differential reporter expression is not due to accumulation of stable fluorescent protein (Fig. 3B). Reporter signal was detected 2 h after Dox injection and was similar to that detected after exogenous addition in culture, indicating that bacteria are exposed to the antibiotic soon after intraperitoneal injection (Fig. 3B). During ATc treatment, reporter expression was significantly higher at the periphery of the microcolony compared to that at the centroid (Fig. 3C). Based on *in vitro* reporter expression (Fig. 2B and D), these results suggest that peripheral bacteria are exposed to inhibitory levels of ∼1 μg/ml tetracyclines, while the interior is exposed to lower subinhibitory concentrations, around 0.1 μg/ml.

### Microcolonies significantly decrease in size after doxycycline treatment.

**FIG 4:**
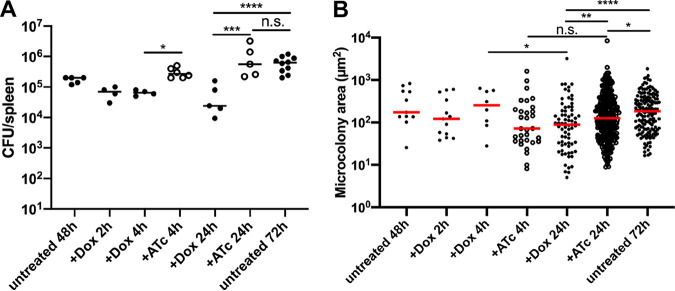
Microcolonies significantly decrease in size after doxycycline treatment. C57BL/6 mice were inoculated i.v. with 10^3^ GFP^+^ P*_tetA_*::*mCherry-ssrA* Y. pseudotuberculosis. Spleens were harvested at 48 h p.i., additional mice were treated at 48 h p.i. with Dox or ATc, and spleens were harvested at the indicated time points posttreatment to quantify CFU and microcolony areas. (A) CFU/spleen quantification from treated or untreated mice. Dots indicate individual mice. Experiments performed in triplicate. (B) Microcolony area (μm^2^) quantification by fluorescence microscopy based on GFP signal, from treated or untreated mice. Dots indicate individual microcolonies. Medians are shown. Statistics: Kruskal-Wallis one-way analysis of variance (ANOVA) with uncorrected Dunn’s test. *, *P* < 0.05; **, *P* < 0.01; ***, *P* < 0.001; ****, *P* < 0.0001; n.s., not significant.

Dox was first detected within host tissues at 2 h posttreatment (Fig. 3B), but it remained unclear how antibiotic administration impacted bacterial growth and viability. To address this, the splenic tissue depicted in Fig. 3 was also harvested to quantify CFU and microcolony areas. CFU decreased 2 h after Dox treatment, and there was a significant reduction in CFU at 4 h of Dox treatment relative to that with ATc treatment (Fig. 4A). The difference in CFU was more pronounced at 24 h posttreatment, where Dox treatment resulted in significantly fewer CFU compared to those with ATc treatment or in untreated mice, and ATc treatment CFU were similar to those in untreated (72-h) mice (Fig. 4A). This suggested the difference in CFU was due to antibiotic activity. There was a significant reduction in microcolony areas between 4 and 24 h of Dox treatment, while microcolony areas increased slightly during ATc treatment, suggesting that Dox activity led to the decrease in microcolony areas (Fig. 4B). Consistent with this, at 24 h posttreatment, Dox treatment resulted in significantly smaller microcolony areas than ATc treatment or those in untreated (72-h) mice (Fig. 4B). ATc treatment also resulted in slightly smaller microcolonies than those in untreated tissue, suggesting there may have been some impact of ATc administration. These results suggest that many bacteria are eliminated following a single Dox treatment, while subsets of viable bacteria also remain within tissues at 24 h after treatment. We believe that smaller microcolonies present at 48 h p.i. are likely eliminated quickly by Dox, and the smaller microcolonies present at 24 h after treatment are remaining cells from larger microcolonies.

### Hmp^+^ cells preferentially survive doxycycline treatment.

**FIG 5:**
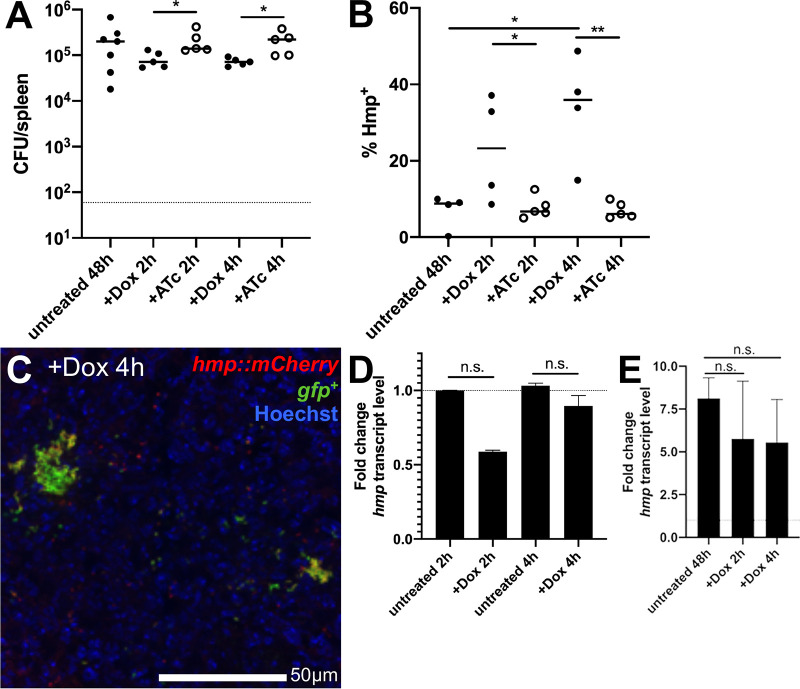
Hmp^+^ cells preferentially survive doxycycline treatment. C57BL/6 mice were inoculated i.v. with 10^3^ GFP^+^ *hmp*::*mCherry* Y. pseudotuberculosis. Spleens were harvested from mice at 48 h p.i. (untreated). Additional mice were treated at 48 h p.i with either Dox or ATc, and spleens were harvested at the indicated time points posttreatment to (A) quantify CFU and (B) quantify the percentage of Hmp^+^ cells by flow cytometry. Dots indicate individual mice. Experiments performed in triplicate. (C) Representative image. (D) WT bacteria were incubated at 37°C for 2 or 4 h, with or without 0.1 μg/ml Dox. RNA was isolated, and qRT-PCR was used to detect *hmp* transcript levels relative to 16S rRNA levels. Fold change was calculated relative to untreated samples; median and range are shown for biological triplicates. (E) C57BL/6 mice were infected with WT Y. pseudotuberculosis, and spleens were harvested from mice at 48 h p.i. (untreated). Additional mice were treated at 48 h p.i with Dox, spleens were harvested at 2 and 4 posttreatment to isolate RNA, and qRT-PCR was performed to detect *hmp* transcript levels relative to 16S rRNA levels. Fold change calculated relative to *hmp* levels in the inoculum. *n* = 3 for untreated groups, *n* = 4 for treated groups; median and range are shown. Statistics: Kruskal-Wallis one-way ANOVA with uncorrected Dunn’s test in panels A, B, and E, and Wilcoxon matched pairs in panel D. *, *P* < 0.05; **, *P* < 0.01; n.s., not significant.

We hypothesized that peripheral bacterial cells stressed by the host immune response may have reduced metabolic activity, and may be predominant within the surviving bacterial population after Dox treatment. The 48-h treatment time point was specifically chosen throughout this study to allow the stressed bacterial subpopulation to develop prior to treatment, to then determine if stress impacts survival of this subpopulation. To determine whether Hmp^+^ cells preferentially survive Dox treatment, we infected C57BL/6 mice intravenously with GFP^+^ *hmp*::*mCherry* Y. pseudotuberculosis and treated mice with Dox or ATc at 48 h p.i., and splenic tissue was harvested at 2 and 4 h posttreatment to quantify CFU and quantify the proportion of Hmp^+^ bacteria by flow cytometry. Early time points posttreatment were used to specifically focus on early survival differences between Hmp-negative (Hmp^−^) and Hmp^+^ cells, and to limit the impact of cell division events that would complicate the analysis. CFU in the spleen were significantly lower with 2 and 4 h of Dox treatment compared to ATc treatment, suggesting that antibiotic activity contributed to decreased CFU (Fig. 5A). Hmp^+^ bacteria were detected by flow cytometry, by gating on the total GFP^+^ bacterial population in the spleen and quantifying the percentage of Hmp^+^ cells before and after treatment. Mice were infected with the GFP^+^ strain in parallel to define the threshold for mCherry^+^ cells. The percentage of Hmp^+^ cells significantly increased after 2 h of Dox treatment compared to that after 2 h of ATc treatment, suggesting that a higher proportion of the Hmp^+^, stressed cells were surviving Dox treatment than the Hmp^−^, unstressed cells, and that this was due to the activity of Dox (Fig. 5B). At 4 h posttreatment, the percentage of Hmp^+^ cells increased significantly with Dox treatment relative to that with ATc treatment and that in untreated mice, again suggesting that Hmp^+^ cells were preferentially surviving drug treatment (Fig. 5B and C). Dox treatment did not impact *hmp* transcript levels *in vitro* (Fig. 5D) or in the mouse spleen (Fig. 5E), indicating that bacteria express *hmp* prior to Dox exposure, and not as a response to treatment. Dox treatment caused a trend toward decreased *hmp* transcript levels both *in vitro* and *in vivo*, although this was not statistically significant. We also confirmed by flow cytometry that exposure to high levels of Dox (1 μg/ml) does not increase the mCherry signal of Y. pseudotuberculosis (Fig. S2), again suggesting that increased *hmp* reporter signal is due to preferential survival of Hmp^+^ cells. Collectively, these results indicate that the stressed Hmp^+^ bacteria at the periphery of microcolonies preferentially survive Dox treatment and that the Dox concentrations reaching the Hmp^−^ interior of microcolonies is sufficient to eliminate a larger proportion of these cells.

### Doxycycline treatment at 48 h p.i. significantly prolongs mouse survival.

**FIG 6:**
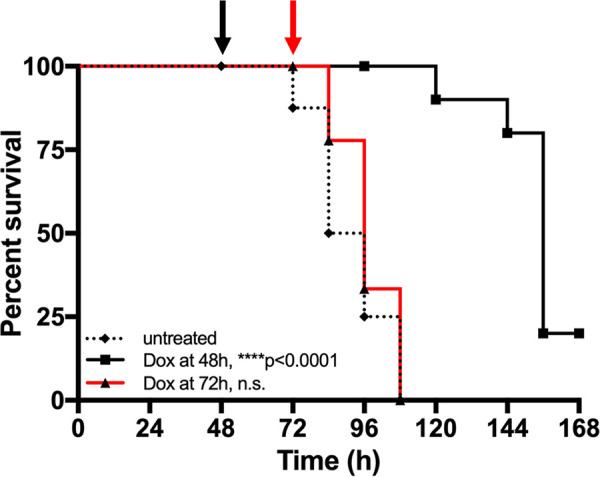
Doxycycline treatment at 48 h significantly prolongs survival. C57BL/6 mice were inoculated i.v. with 10^3^ GFP^+^ WT Y. pseudotuberculosis. Mice were untreated (dotted black line) or injected with Dox at 48 h p.i. (black arrow and line) or 72 h p.i. (red arrow and line), and infection proceeded until mice reached morbidity endpoints. Median survival: untreated, 90 h; Dox at 48 h, 156 h; Dox at 72 h, 96 h. Statistics: log-rank (Mantel Cox) test, compared to untreated group. ****, *P* < 0.0001; n.s., not significant.

Our results suggest that a single dose of Dox is sufficient to eliminate subsets of bacteria in the first 4 h after treatment, but it remained unclear if bacterial numbers continue to drop in the days following treatment and if this single dose is ultimately sufficient to clear infection. Survival curves were performed to determine if a single dose of treatment was sufficient to prolong mouse survival and clear infection. C57BL/6 mice were infected intravenously with the WT Y. pseudotuberculosis GFP^+^ strain, and the infection proceeded until mice reached defined morbidity endpoints. Spleens were plated to confirm morbidity was caused by a high level of CFU (107). Untreated mice had a median survival of 90 h (Fig. 6). One Dox dose at 48 h p.i. was sufficient to significantly prolong mouse survival relative to that of untreated mice; however, treated mice eventually succumbed to the infection, with a median survival of 156 h (Fig. 6). Injection of Dox at 72 h p.i. did not significantly prolong mouse survival (median survival, 96 h), suggesting that the treatment was not effective at this time point. These results suggest that a single dose of Dox is sufficient to significantly prolong mouse survival, but is not sufficient to promote clearance of the infection. The Hmp^+^ cells that initially survive treatment may persist for long periods of time and resume growth when stress and antibiotic concentrations decline.

### Bacterial growth resumes when antibiotic concentrations drop.

**FIG 7:**
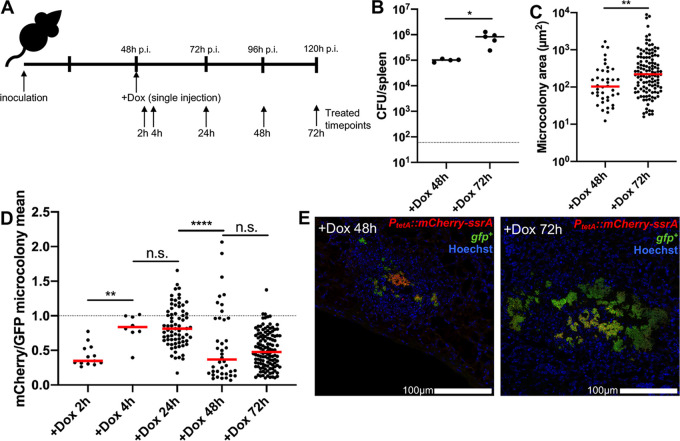
Bacterial growth resumes when antibiotic concentrations decrease. (A) Timeline for mouse infections. C57BL/6 mice were inoculated i.v. with 10^3^ GFP^+^ P*_tetA_*::*mCherry-ssrA* Y. pseudotuberculosis. Mice were treated at 48 h p.i with Dox, and spleens were harvested at the indicated time points posttreatment to quantify CFU and to and quantify microcolony areas and reporter expression by fluorescence microscopy. (B) CFU/spleen quantification. Dots indicate individual mice. Represents data from two experiments. (C) Microcolony area (μm^2^) quantification by fluorescence microscopy, based on GFP signal. Dots indicate individual microcolonies. (D) Quantification of total mCherry reporter signal across microcolonies relative to total GFP signal, as a measure of antibiotic presence and exposure. (E) Representative images. Medians are shown. Statistics: Mann-Whitney in panels B and C and Kruskal-Wallis one-way ANOVA with uncorrected Dunn’s test in panel D. *, *P* < 0.05; **, *P* < 0.01; ****, *P* < 0.0001; n.s., not significant.

The prolonged survival of mice in Fig. 6 suggests that bacterial growth may be restricted for several days after Dox administration, but that eventually bacterial growth resumes and mice succumb to the infection. To determine when bacterial growth resumes within the spleen, C57BL/6 mice were infected with the GFP^+^ P*_tetA_*::*mCherry-ssrA* strain, injected with Dox at 48 h p.i., and tissue was harvested at 48 and 72 h posttreatment to quantify CFU and microcolony areas (Fig. 7A). CFU count remained low at 48 h posttreatment, similar to that at 24 h posttreatment (Fig. 4A); however, there was a significant increase in CFU between 48 and 72 h posttreatment (Fig. 7B). There was also a significant increase in microcolony areas between 48 and 72 h posttreatment (Fig. 7C and E), suggesting that bacterial growth resumes between these time points posttreatment.

To determine if resumed bacterial growth correlates with a change in Dox concentration within the spleen, tissue was harvested at all indicated time points posttreatment to visualize Dox exposure by fluorescence microscopy (Fig. 7A). Microcolonies were detected based on constitutive GFP^+^ signal, and the total mCherry signal across the microcolony was divided by the total GFP to generate a relative exposure value (mCherry mean/GFP mean fluorescent signal) for each microcolony. Using this ratio to detect exposure, there was a significant increase in antibiotic exposure between 2 and 4 h posttreatment with Dox, which remained similar between 4 and 24 h posttreatment (Fig. 7D). Between 24 and 48 h posttreatment, there was a significant decrease in Dox exposure, and this exposure value remained low at 72 h posttreatment (Fig. 7D). This change in Dox exposure based on reporter expression was clearly apparent within microcolonies; many microcolonies retained some Dox reporter signal at 48 h posttreatment, but microcolonies were very large at 72 h posttreatment and had very little remaining reporter signal (Fig. 7E). These results show that Dox concentrations wane in the spleen at 48 h posttreatment, and bacterial growth resumes once the concentration of Dox in the spleen has dropped to subinhibitory levels, ultimately causing the mice to succumb to the infection. Presumably, the stress from host phagocytes has also dissipated at these late time points, permitting the resumption of bacterial growth.

## DISCUSSION

To effectively eliminate infection, antibiotics must be able to penetrate into areas of bacterial replication at high concentrations. Here, we show that tetracyclines diffuse into deep tissue sites and promote the elimination of a majority of the bacterial population within hours of treatment. At 4 h posttreatment, we observed preferential survival of stressed Hmp^+^ cells, and subsequently, tetracyclines accumulate at the periphery of microcolonies between 4 and 48 h posttreatment. The accumulation occurs after we observe preferential survival, which suggests that Dox initially penetrates into microcolonies at doses inhibitory to Hmp^−^ cells. It remains unclear if approximately 100 ng/ml Dox is inhibitory within the host environment, or if initial concentrations of Dox are higher than this approximation. The accumulation of antibiotic is sufficient to prevent bacterial growth, but once the concentration of Dox decreases, bacterial growth resumes, and mice succumb to the infection. These results suggest that penetration of high antibiotic concentrations is not sufficient to eliminate all bacterial cells from deep tissues, due to susceptibility differences of stressed bacterial cells. Unlike granulomas or biofilms, the stressed cells within microcolonies are located at the periphery of bacterial centers and are exposed to high, inhibitory levels of Dox, but this was still not sufficient to eliminate stressed cells.

The data presented here show that Hmp^+^ cells preferentially survive Dox treatment, suggesting that stress caused by NO may be sufficient to slow bacterial growth and reduce antibiotic susceptibility. However, the Hmp^+^ peripheral population is likely exposed to multiple different host-derived stresses, and it also contains a subpopulation of bacteria expressing very high levels of the T3SS (22). For this study, we used *hmp* reporter expression to mark the peripheral population, but we recognize that this is likely a very complex subpopulation, and additional studies will be needed to further characterize the transcriptomic and proteomic profile of this important subpopulation. Additional studies are also needed to truly show that Hmp^+^ cells represent a slow-growing subpopulation with decreased metabolic activity; however, the decreased antibiotic susceptibility of this population suggests this is likely the case (10–12).

During Dox treatment, many bacterial cells survive and are capable of resuming growth once antibiotic concentrations wane (Fig. 7). We hypothesize that smaller microcolonies are initially eliminated and that the surviving cells from larger microcolonies are primarily smaller, broken-down Hmp^+^ pieces. Our data suggest that surviving bacteria may be exposed to Dox concentrations ranging from 0.1 to 1 μg/ml for as long as 48 h, and it will be very interesting to determine how this prolonged exposure impacts the bacterial population. The genes expressed in response to Dox could be potential targets to more effectively eliminate the bacteria population with combination therapeutics. We expect that many of the genes expressed during this long term Dox exposure are protective responses that, if inhibited, would result in a loss of bacterial viability.

Doxycycline treatment has direct effects on the bacterial population, but the release of bacterial products at the site of infection will also impact the host response. Our experiments to detect preferential survival of the Hmp^+^ population were focused on early time points posttreatment, in part because we were interested in early perturbation by Dox, but also because interactions between bacteria and host cells are likely different at later hours posttreatment, as the host immune response becomes activated by release of bacterial ligands. As additional, activated immune cells subsets are recruited to the spleen, we expect the bacterial population to respond to their presence. It would be very interesting to further characterize the bacterial and host response at later time points after Dox treatment, which may provide important clues as to why the host response was not sufficient to completely eliminate the bacterial population.

Antibiotic susceptibility is typically assessed with *in vitro* experiments, and very few studies have focused on the impact of antibiotics in the host environment, where bacteria may respond very differently and may have very different susceptibility patterns. It is critical that we develop tools, such as the fluorescent reporter developed here, to study antibiotic activity, diffusion, and efficacy within mammalian tissues.

## MATERIALS AND METHODS

### Bacterial strains and growth conditions.

The WT Y. pseudotuberculosis strain IP2666 was used throughout (22, 37). For all mouse infection experiments, bacteria were grown overnight (16 h) to the postexponential phase in 2×YT broth (LB with 2× yeast extract and tryptone) at 26°C with rotation. For antibiotic exposure experiments, bacteria were grown in LB overnight (16 h) at 26°C with rotation, subcultured 1:100, and grown at 37°C with rotation in the absence or presence of the indicated concentrations of doxycycline (Dox) or anhydrotetracycline (ATc), until the indicated time points.

### Murine model of systemic infection.

Six- to 8-week old female C57BL/6 mice were obtained from Jackson Laboratories (Bar Harbor, ME). All animal studies were approved by the Institutional Animal Care and Use Committee of Johns Hopkins University. Mice were injected intravenously into the tail vein with 10^3^ bacteria for all experiments. At the indicated time points postinoculation (p.i.), spleens were removed and processed. Intact tissue was stabilized for RNA isolation or fixed for fluorescence microscopy. Tissue was homogenized to quantify CFU and process for flow cytometry. For survival curves, the following morbidity endpoints were used: weight loss of 15% or greater (measured initially and every 24 h p.i.), lethargy, hunched stature, or reduced activity; any of these endpoints qualified mice for euthanasia. Spleens were plated to confirm morbidity was associated with high bacterial CFU counts.

### Generation of reporter strains.

Two of the Y. pseudotuberculosis reporter strains in this study were previously described, WT GFP^+^ and WT GFP^+^ *hmp*::*mCherry* (22). GFP^+^ strains were constructed by transformation with the constitutive GFP plasmid, which expresses GFP from the unrepressed P*_tet_* of pACYC184 (22, 24). The destabilized version of *mCherry* was constructed by fusing an 11-amino-acid *ssrA* tag, with AAV terminal amino acids, between the last coding amino acid of *mCherry* and the stop codon (33, 34). This was inserted downstream of the P*_tac_* isopropyl-β-d-thiogalactopyranoside (IPTG)-inducible promoter in pMMB207Δ267 (38) to quantify mCherry half-life following IPTG induction. A stable WT *mCherry* construct was generated in parallel as a control. The P*_tetA_*::*mCherry* reporter was constructed by amplifying *tetR* through the P*_tetA_* promoter sequence from Tn*10*, fusing this to *mCherry* by overlap extension PCR to replace *tetABC* with *mCherry*, and cloning this into the low-copy pMMB67EH plasmid. P*_tetA_*::*mCherry*-*ssrA* was constructed the same way, by amplifying the *ssrA*-tagged, destabilized *mCherry*.

### Fluorescence detection, plate reader.

Bacteria were washed in phosphate-buffered saline (PBS), resuspended in an equivalent volume of PBS, then added to black-walled, clear-bottomed 96-well plates. Optical density (*A*_600_) was used to approximate cell number; mCherry fluorescence was detected with 560-nm excitation and 610-nm emission, using a Synergy H1 microplate reader (BioTek).

### Determining fluorescent protein half-life.

Overnight cultures (16 h) were grown in LB with 1 mM IPTG. Bacteria were washed 3× in PBS to remove IPTG and resuspended in PBS, and kanamycin (50 μg/ml) was added to inhibit additional protein translation. Bacteria were added to black-walled, clear-bottomed 96-well plates, and were incubated in a Tecan Infinite M200 microplate reader until the indicated time points. Fluorescence reads (580-nm excitation and 620-nm emission) were taken every 5 min. Detection (560-nm excitation/610-nm emission) with a Synergy H1 microplate reader (BioTek) gave equivalent results. Fluorescent protein half-life was calculated from six biological replicates using a one-phase exponential decay equation in Prism software.

### Fluorescence microscopy: host tissues.

Spleens were harvested and immediately fixed in 4% paraformaldehyde (PFA) in PBS for 3 h. Tissues were frozen-embedded in SubXero freezing medium (Mercedes Medical) and cut by cryostat microtome into 10-μm sections. To visualize reporters, sections were thawed in PBS, stained with Hoechst stain at a 1:10,000 dilution, and washed in PBS, and coverslips were mounted using ProLong Gold (Life Technologies). Tissue was imaged as described above, with an Apotome.2 system (Zeiss) for optical sectioning.

### Quantitative reverse transcription-PCR to detect bacterial transcripts in broth-grown cultures.

Bacterial cells were grown in the presence or absence of 0.1 μg/ml Dox for the indicated time points, pelleted, and resuspended in buffer RLT (Qiagen) plus β-mercaptoethanol, and RNA was isolated using the RNeasy kit (Qiagen). DNA contamination was eliminated using a DNA-free kit (Ambion). RNA was reverse transcribed using Moloney murine leukemia virus (MMLV) reverse transcriptase (Invitrogen) in the presence of the RNase inhibitor RNaseOUT (Invitrogen). Approximately 30 ng cDNA was used as a template in reactions with 0.5 μM forward and reverse primers (22) and SYBR green (Applied Biosystems). Control samples were prepared that lacked MMLV, to confirm that DNA was eliminated from samples. Reactions were carried out using the StepOnePlus real-time PCR system, and relative comparisons were obtained using the threshold cycle (ΔΔ*C_T_* or 2^−Δ^*^CT^*) method (Applied Biosystems). Kits were used according to the manufacturers’ protocols.

### Quantitative reverse transcription-PCR to detect bacterial transcripts from mouse tissues.

Spleens were harvested and immediately submerged in RNALater solution (Qiagen). Tissue was homogenized in buffer RLT plus β-mercaptoethanol, and RNA was isolated using the RNeasy kit (Qiagen). Bacterial RNA was enriched following depletion of host mRNA and rRNA from total RNA samples, using the Microb*Enrich* kit (Ambion). Kits were used according to manufacturers’ protocols; DNA digestion, reverse transcription, and quantitative reverse transcription-PCR (qRT-PCR) were performed as described above.

### Flow cytometry.

Homogenized tissue was treated with collagenase D and DNase I in PBS plus 0.2 mM EDTA to dissociate host cells from bacteria. Host cells were lysed with eukaryotic lysis buffer (5 mM EDTA, 0.1% NP-40, 0.15 M NaCl, and 1 M HEPES), debris were settled out of solution, and supernatant was collected, pelleted, and fixed in 4% PFA overnight at 4°C. Bacteria were detected using GFP signal and forward scatter (FSC; size), and 1,000 bacterial events were collected per tissue, using a BD FACSCalibur. For analysis, bacterial cells were gated based on GFP signal, and GFP^+^ bacterial controls lacking mCherry constructs (prepared and collected in parallel) were used to identify mCherry^+^ cells.

### Image analysis.

Volocity image analysis software was used to quantify microcolony areas and fluorescence as previously described (22). Zen 2 colocalization software was used to generate an intensity dot plot for pixels within an image (Fig. 2F) with a corresponding pseudocolored image; each quadrant of the dot plot corresponds with a highlighted pseudocolor. Bacterial cells defined the GFP^+^ threshold; within this threshold, the pixels with the highest mCherry signal intensity (66%) were considered mCherry^high^, and pixels with the lowest (34%) mCherry signal intensity were considered mCherry^low^. ImageJ was used to quantify the signal intensity of each channel at the centroid and periphery of each microcolony to generate relative signal intensities of fluorescent reporters, as previously described (22). Thresholding was used to define the total area and centroid of each microcolony, and 0.01 pixel^2^ squares were selected to calculate values. Peripheral measurements depict bacteria in contact with host cells; peripheral cells are highlighted outside dotted lines in Fig. 2E, Fig. 3E and F.

10.1128/mBio.00901-20.3 TEXT S1 Supplemental Materials and Methods for fluorescence microscopy: bacteria. To visualize individual bacterial cells, samples were pelleted, resuspended in 4% paraformaldehyde (PFA), and incubated overnight at 4°C for fixation. PFA was removed and bacteria were resuspended in phosphate-buffered saline (PBS) prior to imaging. Agarose pads were prepared to immobilize bacteria for imaging, by solidifying a thin layer of 25 μl 1% agarose in PBS between a microscope slide and coverslip. Once solidified, coverslips were removed, bacteria were added, coverslips were replaced, and bacteria were imaged with the 63× oil immersion objective, using a Zeiss Axio Observer 7 (Zeiss) inverted fluorescent microscope with XCite 120LED boost system and an Axiocam 702 mono camera (Zeiss). Volocity image analysis software was used to specifically select individual bacterial cells and quantify the fluorescent signal associated with each cell. Download Text S1, DOCX file, 0.01 MB. Copyright © 2020 Ramirez Raneses et al. 2020 Ramirez Raneses et al. This content is distributed under the terms of the Creative Commons Attribution 4.0 International license.
