# Supplementary figures and images for: Subpopulations of Stressed Yersinia pseudotuberculosis Preferentially Survive Doxycycline Treatment within Host Tissues
Source: mBio. 2020 Aug 4;11(4):e00901-20. doi: 10.1128/mBio.00901-20 (PMC7407081; doi:10.1128/mBio.00901-20)

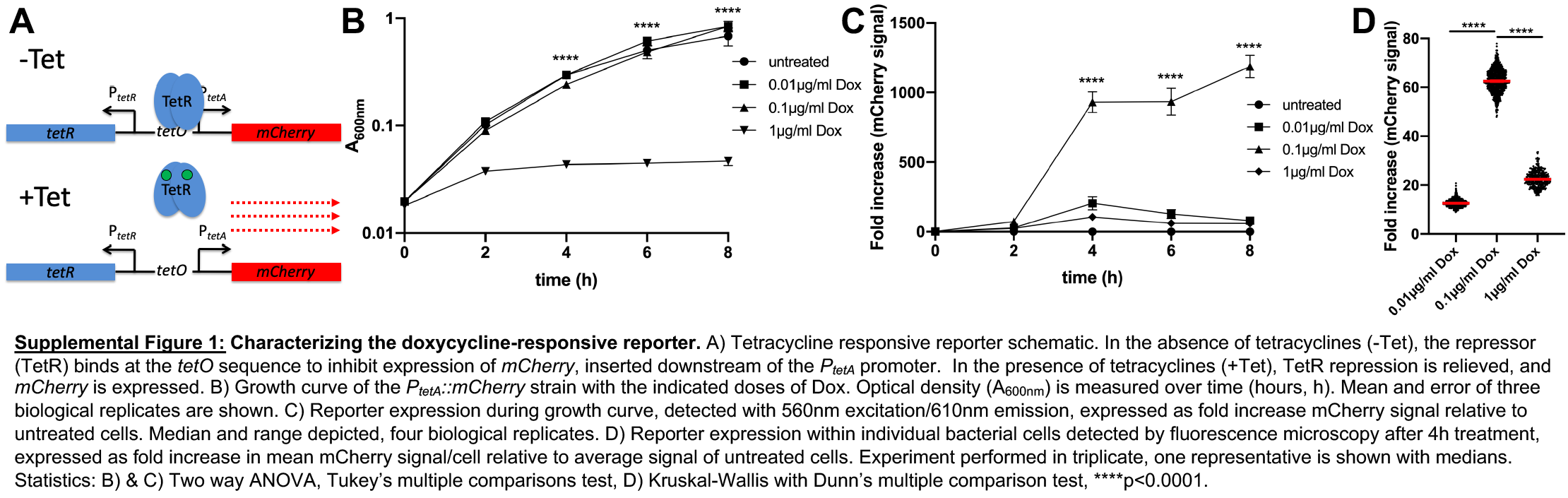

Supplement: FIG S1 [file mBio.00901-20-sf001.tif]

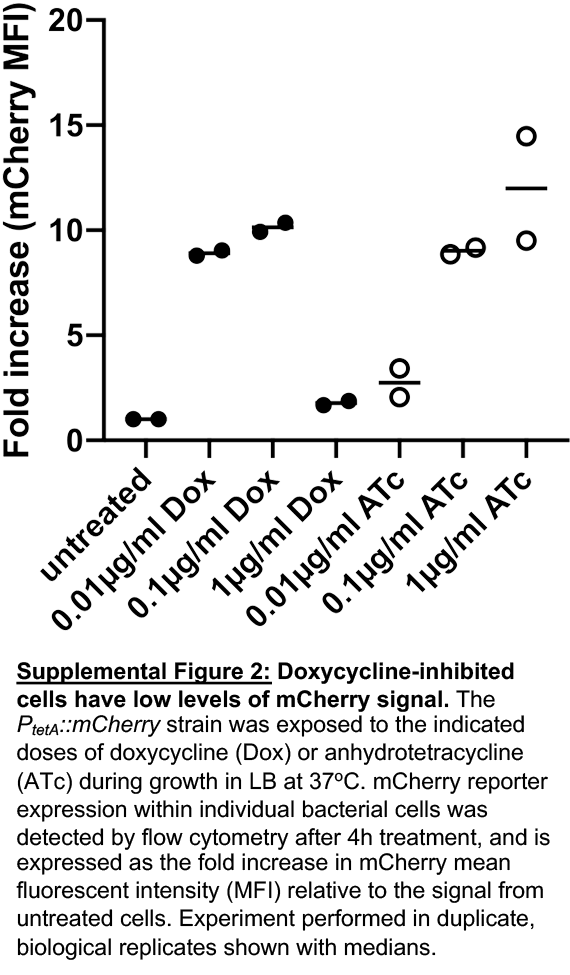

Supplement: FIG S2 [file mBio.00901-20-sf002.tif]
