# Supplementary material for: Subpopulations of Stressed Yersinia pseudotuberculosis Preferentially Survive Doxycycline Treatment within Host Tissues
Source: mBio. 2020 Aug 4;11(4):e00901-20. doi: 10.1128/mBio.00901-20 (PMC7407081; doi:10.1128/mBio.00901-20)
Supplement: TEXT S1 [file mBio.00901-20-s0001.docx]

**Supplemental Materials.**

**Figure Legends:**

**Supplemental Figure 1: Characterizing the doxycycline-responsive reporter.** A) Tetracycline responsive reporter schematic. In the absence of tetracyclines (-Tet), the repressor (TetR) binds at the *tetO* sequence to inhibit expression of *mCherry*, inserted downstream of the *P_tetA_* promoter. In the presence of tetracyclines (+Tet), TetR repression is relieved, and *mCherry* is expressed. B) Growth curve of the *P_tetA_::mCherry* strain with the indicated doses of Dox. Optical density (A_600nm_) is measured over time (hours, h). Mean and error of three biological replicates are shown. C) Reporter expression during growth curve, detected with 560nm excitation/610nm emission, expressed as fold increase mCherry signal relative to untreated cells. Median and range depicted, four biological replicates. D) Reporter expression within individual bacterial cells detected by fluorescence microscopy after 4h treatment, expressed as fold increase in mean mCherry signal/cell relative to average signal of untreated cells. Experiment performed in triplicate, one representative is shown with medians. Statistics: B) & C) Two way ANOVA, Tukey’s multiple comparisons test, D) Kruskal-Wallis with Dunn’s multiple comparison test, ****p<0.0001.

**Supplemental Figure 2:** **Doxycycline-inhibited cells have low levels of mCherry signal.** The *P_tetA_::mCherry* strain was exposed to the indicated doses of doxycycline (Dox) or anhydrotetracycline (ATc) during growth in LB at 37^o^C. mCherry reporter expression within individual bacterial cells was detected by flow cytometry after 4h treatment, and is expressed as the fold increase in mCherry mean fluorescent intensity (MFI) relative to the signal from untreated cells. Experiment performed in duplicate, biological replicates shown with medians.

**Supplemental Material and Methods:**

*Fluorescence microscopy: bacteria.* To visualize individual bacterial cells, samples were pelleted, resuspended in 4% paraformaldehyde (PFA) and incubated overnight at 4^o^ C for fixation. PFA was removed and bacteria were resuspended in PBS prior to imaging. Agarose pads were prepared to immobilize bacteria for imaging, by solidifying a thin layer of 25µl 1% agarose in PBS between a microscope slide and coverslip. Once solidified, coverslips were removed, bacteria were added, coverslips were replaced, and bacteria were imaged with the 63x oil immersion objective, using a Zeiss Axio Observer 7 (Zeiss) inverted fluorescent microscope with XCite 120 LED boost system and an Axiocam 702 mono camera (Zeiss). Volocity image analysis software was used to specifically select individual bacterial cells and quantify the fluorescent signal associated with each cell.
